# Determinants of spousal physical violence against women in Zambia: a multilevel analysis

**DOI:** 10.1186/s12889-023-15927-x

**Published:** 2023-05-23

**Authors:** Million Phiri, Sibongile Namayawa, Bruce Sianyeuka, Palver Sikanyiti, Musonda Lemba

**Affiliations:** 1 Department of Population Studies, School of Humanities and Social Sciences, University of Zambia, Lusaka, Zambia; 2 Demography and Population Studies Programme, Schools of Public Health and Social Sciences, University of the Witwatersrand, Johannesburg, South Africa; 3 Zambia Statistics Agency, Lusaka, Zambia

**Keywords:** Women, Physical spousal violence, Intimate partner violence, Multilevel analysis, Zambia

## Abstract

**Background:**

Violence against women and girls is a major public health issue, a violation of human rights, and is linked to a number of harmful effects on one’s physical, mental, sexual, and reproductive health. Studies conducted in other parts of sub-Saharan Africa (SSA) suggest that there is an association between contextual factors and experience of intimate partner violence. However, in Zambia, this association is not well documented. Thus, this study was conducted to examine how individual and community-level characteristics influence spousal violence against women in Zambia.

**Methods:**

Data from the most recent Zambia Demographic and Health Survey conducted in 2018 was used. A sample of 7,358 ever-married women aged 15–49 years was used in the analysis. Two level multilevel binary logistic regression models were employed to examine the association between individual and contextual-level factors and experience of spousal violence.

**Results:**

The prevalence of spousal physical violence against women in Zambia was 21.1% [95% CI, 19.8, 22.5]. Women aged 15–19 [aOR = 2.36, 95% CI = 1.34–4.14] and 20–24 [aOR = 2.11, 95% CI = 1.38–3.22], who did not own mobile phone [aOR = 1.36, 95% CI = 1.10–1.69], and had low decision making autonomy [aOR = 1.24, 95% CI = 1.01–1.54] were more likely experience spousal physical violence. Furthermore, communities which had a low proportion of women with decision making power [aOR = 1.66, 95% CI = 1.26–2.19] were more likely experience spousal physical violence. Additionally, women whose partners’ drank alcohol [aOR = 2.81, 95% CI = 2.30–3.45] and those whose partners exhibited jealous behaviour [aOR = 2.38, 95% CI = 1.88–3.21] were more likely to experience spousal physical violence.

**Conclusion:**

Both individual and community-level factors influenced spousal physical violence in Zambia. Integrating community level factors when designing interventions to address gender-based would be key to reduce women’s vulnerability to gender based violence in the country. There is need to re-evaluate and re-strategize current strategies being implemented to address gender based violence in the country to make them context specific.

## Introduction

The global epidemic of violence against women and girls is a major public health issue, a violation of human rights, and is linked to a number of harmful effects on one’s physical, mental, sexual, and reproductive health [1–5]. Despite many international efforts to reduce violence against women and girls, especially in sub-Saharan Africa (SSA), the negative effects it has on the health and the wellbeing of women’s and girls’ continue unabated [2, 6, 7]. Any behaviour in an intimate relationship that hurts the other person physically, psychologically, or sexually is defined to as intimate partner violence (IPV) [8]. IPV is one of the most common forms of violence against women and girls. It includes physical acts such as slapping, striking, kicking, and beating as well as unwanted sexual acts, mental abuse, and use of abusive behaviors on the side of an intimate partner [9, 10].

The World Health Organization (WHO) estimates that, one-third of women around the world have experienced some form of violence (physical or sexual) from a partner or non-partner at some point in their lifetime [10, 11]. Recent studies on IPV reveal that, globally, 13 to 61% of victims have experienced physical abuse from a partner, 4 to 49% have experienced severe physical abuse from a partner, 6 to 59% have experienced sexual abuse from a partner at some point in their lives, and 20 to 75% have experienced one emotionally abusive act in their lifetime [8, 12, 13].

Women who have a history of experiencing intimate partner violence can be at risk to many diseases and conditions [14–16]. Major effects of IPV on women include drug and alcohol abuse, eating and sleeping disorders, physical inactivity, low self-esteem, post-traumatic stress disorder, smoking, and self-harm, while major risks for children include anxiety, depression, poor academic performance, and unfavourable health outcomes [14–19]. Previous studies on IPV conducted in different countries in SSA including Zambia have identified various factors like place of residence, maternal age, level of education, wealth status, employment status, number of children, media exposure, women’s decision making power, gender norms and type of marriage as being associated with experience of IPV [2, 5, 9, 20–26].

However, no evidence showed studies conducted in Zambia at a national or subnational level to examine the influence of contextual-level factors on experience of spousal physical violence via multilevel analysis. Studies conducted in other countries found that communities with a high proportion of empowered women, rich households and education women had less exposure to spousal violence while those with high acceptance of IPV norms had higher prevalence of intimate partner violence [27–31]. Studying both individual and contextual factors that are associated with spousal violence would be key to inform designing of evidence- based interventions to address differing community needs. Therefore, the results of this current analysis will help policymakers implement context-specific interventions aimed at reducing intimate partner violence in the country. Moreover, the study findings will also provide evidence to give direction for multisectoral bodies to focus on different strategies to eliminate the vice and overcome its negative consequences. Therefore, the objective of this study is to examine individual and contextual factors that are associated with experience of spousal physical violence against women in Zambia using multivariate mixed effect analysis based on the most recent 2018 Zambia Demographic and Health Survey.

## Methods and data

### Data source

**Fig. 1:**
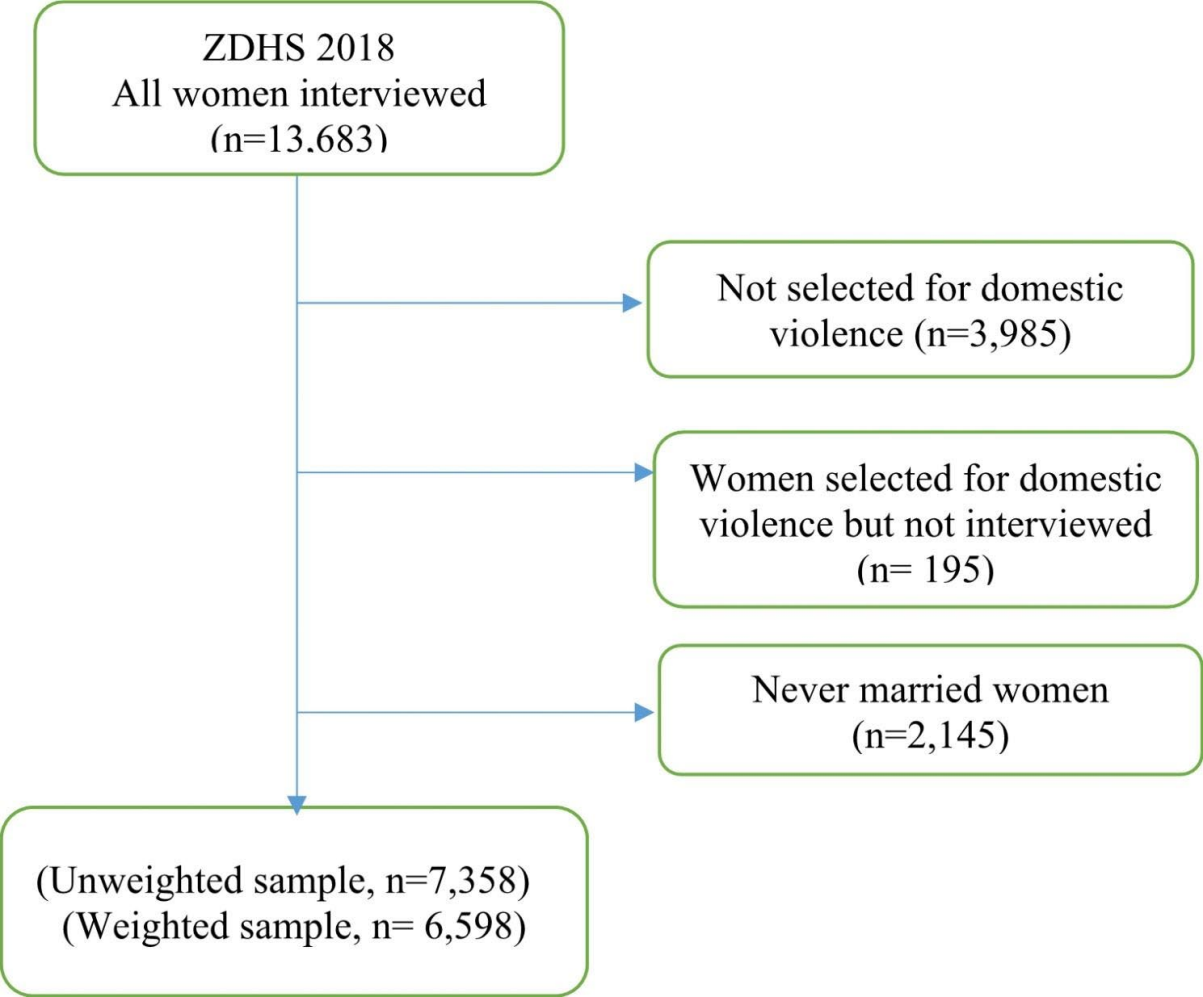
Description of sample derivation criteria

Data from the Zambia Demographic and Health Survey (ZDHS) conducted in 2018 was used in this study. Specifically, the study used the women’s dataset which contains the responses of women of reproductive ages 15–49 years. The Demographic and Health Survey is a nationwide survey that is carried out across many low-and middle-income countries every five-years [32] to collects data on a number of indicators such as family planning, maternal health, child health and domestic violence. The DHS has been an essential source of health data on issues surrounding maternal health in developing countries as it gathers data on several maternal health and sexual and reproductive health issues. The DHS uses two-stage sampling to select enumeration areas (EAs) in the first stage and households in the second stage. For this analysis, a sample of 7,358 (weighted = 6,598) ever-married women aged 15–49 years who completed the domestic violence module and had complete information on the outcome variable of interest (physical violence) were included in the analysis. The selection criteria for the analytical sample size is described Fig. 1.

### Study measurements

#### Outcome measure

The outcome variable for this study was “intimate partner violence” focusing on physical violence. Most studies on gender based violence in SSA have shown that physical violence is the most common abuse inflicted on women [2, 6, 33, 34]. Intimate partner violence was measured in two forms in the DHS. The first form measured the proportion of women who ever experienced intimate partner violence and the second form the experience of violence in the past 12 months preceding the survey. In this study, we used a more recent measure of violence. Thus, our dependent variable was experience of physical violence in the past 12 months preceding the DHS. In DHS definition; physical violence constituted any of the following acts: pushed, slapped, punched with a fist, kicked, strangled, threatened by knife and arm twisted. Thus, experiencing any of the seven acts of physical violence qualified one to be classified as having experienced physical violence [35]. The dependent variable was categorised as binary with “1” representing experience of physical spousal violence and “0” representing non-experience of spousal physical violence.

#### Independent correlates

Based on exiting literature [36–40], the following explanatory variables were selected: age of a woman, education, wealth status, residence, employment status, age at first marriage, marital status, parity, owner of mobile phone, exposure to family planning messages, exposure of radio/television, newspaper, and desired family size. These variables were categorised at individual and contextual-levels.

#### Individual level factors

Based on existing literature [2, 6, 33, 34], a number of independent correlates were identified, these included: age of a woman; level of education attained by a woman; wealth status of household; age at first marriage; ownership of mobile phone; and decision making power at household level. The woman’s independent correlates were classified as follows: age categorized as [15–49]; education level (none, primary, secondary and higher); household wealth status (poor, middle and rich); employment status (not working and working); age at first marriage (less than 15, 15–19, 20 year or above); ownership of mobile phone (yes/no); and woman decision making autonomy (low/high). The variable, woman decision making autonomy, was a composite variable created from a set of three variables captured in the DHS (decision about health, decision about large household purchases and decision about visit to family or relatives). Media exposure was also created as a composited variable from the 3 DHS variables, frequency of listening to radio, frequency of watching television and frequency of reading newspapers. The indicator was measured as a binary response (yes/no). The study also identified variables that relate to a woman’s partner or husband. These included: whether partner has other wives; whether partner drinks alcohol; whether partner exhibited jealous behaviour; and whether partners accuses a woman of unfaithfulness. The correlates related to a partner or husband were classified as follows: partner’s level of education (none, primary, secondary and higher); whether partner has other wives (yes/no); whether partner drinks alcohol (yes/no); whether partner exhibited jealous behaviour (yes/no) and whether partners accuses a woman of unfaithfulness (yes/no).

#### Community-level factors

The aggregation of sociodemographic characteristics, access and behaviour-related factors from individual-level to contextual-level was done to study variables at the community level. These variables were chosen based on their significance in previous studies [41, 42]. The primary sampling unit (i.e., cluster) of the ZDHS survey was defined as a community [43]. The proportion of women in the cluster was determined by their place of residence, household wealth status, women’s education, employment status, decision making autonomy at household level, age at first marriage, and exposure to media. Percentiles were categorised into three levels for simple interpretation (low, middle, and high). Contextual-level variables were categorised as place of residence (rural, urban). Socioeconomic factors included the following community wealth (low, medium, high); community education (low, medium, high); and community employment status (low, medium, high). Gender norms included: community woman decision making autonomy (low, medium, high); and access to information, community exposure to media (low, medium, high).

### Statistical analysis

Data analysis was done at three levels, descriptive, bivariate and multi-level logistic regression. All analyses were performed using Stata version 17 software. At descriptive level, proportional distributions of outcome indicators were determined. At bivariate level, cross-tabulations with chi-square tests were used to assess the association between experience of spousal physical violence and selected independent variables. To assess the effects of several individual and community level factors on experience of spousal violence among the ever-married women in Zambia, a two-level multi-level binary logistic regression model was applied on 2018 DHS survey. The “melogit” command was used in Stata software to perform a two-level’ multilevel analysis.

Adjusted odds ratios (aOR) with corresponding 90%, 95% and 99% confidence intervals (CI) were reported in the presentation of results. Four multilevel logistic models were estimated. Model 0 only included the outcome variable. Model 1 included the individual level variables and model 2 included only community level variables, while Model 3 had both the individual and community level variables.

In terms of variances used to understand the variations of relationships between communities and the relative effect of community-level variables, the intraclass correlation coefficient (ICC) was used. The ICC provides information on the share of variance at each levelTo explain the heterogeneity in the probabilities of early marital experience, the Proportional Change in Variance (PCV) was computed for each model in comparison to the empty model [44–46].

The goodness of fit of the models were evaluated using the Akaike Information Criteria (AIC) and a model with a lower AIC was considered to be a better fit for the data. Wanatabe – Aikake Information Criteria (WAIC) were used to compare models and measure goodness of fit [45, 46]. All variables with level of level of significance of less than 0.2 were included in multivariate multilevel analysis.

### Ethical approval

The data analysed in this study is available in the public domain at (https://dhsprogram.com/) Permission to the data was obtained from the DHS program. All data used did not contain any identifying information. The original Zambia DHS 2018 Biomarker and survey protocols were approved by Tropical Disease and Research Center (TDRC) and the Research Ethics Review Board of the Centers for Disease Control and Prevention (CDC) Atlanta. Thus, all data collection methods were carried out in accordance with relevant ethical guidelines and regulation**s**. The DHS protocols ensured that all participants older than 18 years who were enrolled in the DHS give their informed consent during enumeration. Additionally, parents or guardians of all participants aged 15 to 17 gave informed consent before the legal minors were asked for their assent.

## Results

### Description of sample characteristics

The findings regarding the background characteristics of the study sample are summarized in Table 1. Slightly close to 1 in 5 (19.8%) of the study sample were within 25–29 years while about 5% were in the age range 15–19 years. Most (50.5%) of the study respondents had primary level education while 5% had attained tertiary level education. About 41% and 40% of them were from rich and poor households, respectively. The majority (58.1%) of the respondents were married when they were in the age range 15–19 years and about 10% were married at an age below 15 years. The study sample was also made up of close to 62% of women who were in employment. The largest proportion of the sampled women (35.8%) had parity of two or three children at the time of the survey and more than half (58.5%) of them were living in rural areas. Majority of women owned a mobile phone (53.9%).

**Table 1:** Percent distribution of background characteristics of ever-married women (15–49 years), 2018 DHS, Zambia

Background Characteristics	DHS 2018 (N = 7,358)	
	Number	Percent
**Age**		
15–19	319	4.8
20–24	1,160	17.6
25–29	1307	19.8
30–34	1210	18.3
35–39	1144	17.3
40–44	835	12.7
45–49	624	9.5
**Residence**		
Urban	2740	41.5
Rural	3858	58.5
**Education level**		
None	673	10.2
Primary	3334	50.5
Secondary	2258	34.2
Higher	332	5.0
**Wealth status**		
Poor	2642	40.0
Middle	1269	19.2
Rich	2687	40.7
**Age at first marriage**		
Less than 15	656	9.9
15–19	3832	58.1
20+	2110	32.0
**Employment status**		
Not working	2516	38.2
Working	4073	61.8
**Living children**		
Zero –One	285	4.3
Two-Three	2365	35.8
Four-Five	1971	29.9
Six or more	1976	30.0
**Ownership of mobile phone**		
No	3058	46.3
Yes	3540	53.7

### Prevalence of spousal physical violence among ever-married women

Table 2 shows the distribution of results for experience of spousal physical violence across different individual and household level factors. With regard to the individual-level factors, the highest prevalence of spousal physical violence was found among women aged 20–24 (24.4%), those not working (21.5%), those who were married when in the age group 15–19 (22.6%), those who did not own a mobile phone (24.4%), and those with primary or no education (22.9% and 21.5%, respectively). Furthermore, women who had no decision autonomy (24.4%) and those with no exposure to media (23.0%) had higher rates of experiencing spousal physical violence. In terms of household level factors, the highest prevalence of spousal physical violence was found among women in urban areas (21.2%) and those in the poor wealth quintile (22.9%). The chi-square test of independence results revealed that apart from residence and woman’s employment status, all the independent correlates were statistically associated with experience of spousal physical violence among women.

**Table 2:** Percent distribution of experience of spousal physical violence among ever-women (15–49 years) by background characteristics, 2018 DHS, Zambia

Background Characteristics	DHS 2018 (N = 7,358)		
	Experienced spousal physical violence	Did not experience spousal physical violence	p-value
**Age**			p < 0.05*
15–19	73 (22.9)	245 (77.1)	
20–24	283 (24.4)	876 (75.6)	
25–29	291 (22.3)	1015 (77.7)	
30–34	269 (22.2)	939 (77.7)	
35–39	219 (19.2)	924 (80.8)	
40–44	152 (18.2)	682 (81.8)	
45–49	102 (16.4)	521 (83.6)	
**Residence**			**p > 0.05**
Urban	581 (21.2)	2158 (78.8)	
Rural	810 (21.0)	3047 (79.0)	
**Education level**			P < 0.01**
None	144 (21.5)	528 (78.5)	
Primary	762 (22.9)	2571 (77.1)	
Secondary	452 (20.0)	1806 (80.0)	
Higher	32 (9.8)	299 (90.2)	
**Wealth status**			p < 0.01**
Poor	605 (22.9)	2036 (77.1)	
Middle	285 (22.5)	983 (77.5)	
Rich	501 (18.7)	2185 (81.3)	
**Age at first marriage**			p < 0.01**
Less than 15	147 (22.5)	508 (77.5)	
15–19	864 (22.6)	2967 (77.4)	
20+	379 (18.0)	1730 (82.0	
**Woman’s employment status**			**p > 0.05**
Not working	540 (21.5)	1975 (78.5)	
Working	851 (20.9)	3221 (79.1)	
**Ownership of mobile phone**			p < 0.001***
No	746 (24.4)	2311 (75.6)	
Yes	645 (18.2)	2894 (81.8)	
**Exposure to media**			p < 0.05*
No	597 (23.0).	2000 (77.0)	
Yes	795 (19.8)	3206 (80.1)	
**Woman decision autonomy**			p < 0.001***
No	840 (24.4)	2605 (75.6)	
Yes	551 (17.5)	2600 (82.5)	
**Partner drinks alcohol**			p < 0.001***
No	551 (13.4)	3556 (86.6)	
Yes	840 (33.8)	1649 (66.2)	
**Partner has more than one wife**			p < 0.000***
No	934 (19.8)	3780 (80.2)	
Yes	181 (29.7)	431 (70.3)	
**Partner jealous**			p < 0.000***
**No**	262 (9.5)	2509 (90.5)	
**Yes**	1121 (29.7)	2659 (70.3)	
**Partner accuses wife of being unfaithful**			p < 0.001***
No	623 (13.7)	3942 (86.3)	
Yes	759 (37.8)	1247 (62.2)	
**Total**	**21.1**	**78.9**	

*** *p* < 0.001; ** = *p* < 0.01; * = *p* < 0.05

### Determinants of experience of spousal physical violence among ever-married women

The study found statistically significant associations between individual and community-level factors of experiencing spousal physical violence. Specifically, women in the age group 15–19 years [aOR = 2.36, 95% CI = 1.34–4.14] and 20–24 years [aOR = 2.11, 95% CI = 1.38–3.22] had higher odds of experiencing spousal physical violence compared to older women in the age group 45–49 years. Although not significant, women who had tertiary level education [aOR = 1.08, 95% CI = 0.52–2.23] were less likely to experience spousal physical violence compare to those with no education. Further, results show that women who did not own mobile phones [aOR = 1.36, 95% CI = 1.10–1.69] had higher odds of experiencing spousal physical violence compared to those with mobile phones. Women who had low decision making autonomy [aOR = 1.24, 95% CI = 1.01–1.54] were equally more likely to experience spousal physical violence compared to those with high decision making autonomy. Women whose partners drank alcohol [aOR = 2.82, 95% CI = 2.30–3.45] or whose partners had more than one wife [aOR = 1.50, 95% CI = 1.18–1.90] or whose partners’ were jealous [aOR = 2.38, 95% CI = 1.88–3.02] had higher odds of experiencing spousal physical violence. Similarly, women whose partner’s accused them of being unfaithful [aOR = 2.63, 95% CI = 2.18–3.22] were more likely to experience spousal physical violence compared to their defined counterparts (Table 3).

Regarding to the influence of the community-level factors on experience of spousal violence against women, the findings indicate that ever-married women who belonged to communities that had a high percentage of women who had low decision making autonomy [aOR = 1.66, 95% CI = 1.26–2.19] were more likely to experience spousal physical violence compared to those from communities with low proportion of women with decision making autonomy. Although not statistically significant, communities with high proportion of working women [aOR = 0.90, 95% CI = 0.70–1.16] were less likely to experience spousal physical violence compared to communities with low proportion of employed women. Conversely, communities with a high proportion of women who were married at a young age [aOR = 1.24, 95% CI = 0.96–1.59] were more likely to experience spousal physical violence compared to those from communities with low proportion of women marrying at a young age (Table 3).

Measures of variation for experience of spousal physical violence are presented in Table 3. In the null model, the use of multilevel modelling was justified by the significant variation in prevalence of experience of physical spousal violence (σ^2^ = 0.27, 95% CI 0.19–0.38). Model 0 showed that 7.6% of the total variation in experience of spousal physical violence was attributed to the variance between clusters (ICC = 0.076). The between-cluster variance showed a decrease from 7.6 to 5.9% from Model 0 to Model 1 (individual level factors only). From Model I, the ICC further reduced to 5.4% in Model II (model with community level factors only), and decreased further to 4.1% in the full model (Model III), where all the independent correlates and community level factors were considered. Additionally, 48.1% of the variance in the odds of experiencing spousal violence across communities were explained by both individual and community-level factors, as indicated by the Proportional Change in Variance (PCV) in model IV. Model III which had a lower AIC was considered as the model of best fit (Log-likelihood = − 2167.4, AIC = 4408.8).

**Table 3:** Multilevel parameter estimates and odds of experience of spousal physical violence among ever-married women aged [15–49] by individual and community level characteristics, ZDHS 2018 (N = 7,358)

Variables	Model 0	Model I	Model II	Model III
		aOR (95%CI)	aOR (95%CI)	aOR (95%CI)
**Individual level factors**				
**Age**				
15–19		2.35** [1.33, 4.14]		2.36** [1.34, 4.14]
20–24		2.17*** [1.42, 3.30]		2.11** [1.38, 3.22]
25–29		1.81** [1.21, 2.70]		1.78* [1.19, 2.66]
30–34		1.79* [1.19, 2.70]		1.74* [1.15, 2.63]
35–39		1.34 [0.86, 2.09]		1.32 [0.84, 2.01]
40–44		1.43 [0.88, 2.31]		1.43 [0.88, 2.32]
45–49		**1**		**1**
**Education level**				
None		**1**		**1**
Primary		1.15 [0.85, 1.56]		1.18 [0.88, 1.60]
Secondary		1.21 [0.83, 1.76]		1.23 [0.84, 1.79]
Higher		0.98 [0.48, 2.03]		1.08 [0.52, 2.23]
**Partner’s education**				
None		**1**		**1**
Primary		1.02 [0.73, 1.42]		1.02 [0.73, 1.42]
Secondary		0.87 [0.62, 1.23]		0.86 [0.61, 1.22]
Higher		0.62 [0.33, 1.15]		0.63 [0.34, 1.18]
**Household wealth status**				
Poor		**1**		**1**
Middle		1.09 [0.86, 1.37]		0.93 [0.72, 1.19]
Rich		1.09 [0.82, 1.45]		0.82 [0.56, 1.20]
**Age at first marriage**				
Less than 15		**1**		**1**
15–19		1.18 [0.88, 1.58]		1.20 [0.90, 1.61]
20+		1.18 [0.87, 1.60]		1.22 [0.90, 1.165]
**Employment status**				
Not working		**1**		**1**
Working		0.92 [0.74, 1.13]		0.93 [0.74, 1.17]
**Ownership of mobile phone**				
No		1.34* [1.07, 1.67]		1.36* [1.10, 1.69]
Yes		**1**		**1**
**Exposure to media**				
No		0.98 [0.78, 1.23]		0.98 [0.78, 1.24]
Yes		**1**		**1**
**Woman decision making autonomy**				
Low		1.37** [1.13, 1.65]		1.24* [1.01, 1.54]
High		**1**		**1**
**Partner drinks alcohol**				
No		**1**		**1**
Yes		2.83*** [2.31, 3.47]		2.82***[2.30, 3.45]
**Partner has more than one wife**				
No		**1**		**1**
Yes		1.48** [1.16, 1.88]		1.50** [1.18, 1.90]
**Partner jealous**				
No		**1**		**1**
Yes		2.40*** [1.89, 3.05]		2.38***[1.88, 3.02]
**Partner accuses wife of being unfaithful**				
**No**		**1**		**1**
**Yes**		2.65*** [2.18, 3.22]		2.65***[2.18, 3.22]
**Contextual variables**				
**Place of residence**				
Urban			**1**	**1**
Rural			0.64** [0.48, 0.84]	0.83 [0.59, 1.15]
**Community education**				
Low			0.93 [0.69, 1.26]	0.83 [0.53, 1.20]
Moderate			1.16 [0.91, 1.48]	1.13 [0.83, 1.50]
High			**1**	1
**Community wealth status**				
Low			**1**	**1**
Moderate			0.88 [0.68, 1.13]	1.34 [0.99, 1.81]
High			0.67* [0.47, 0.95]	1.20 [0.78, 1.87]
**Community employment**				
Low			**1**	**1**
Moderate			0.96 [0.81, 1.15]	1.12 [0.91, 1.38]
High			0.95 [0.78, 1.17]	0.90 [0.70, 1.16]
**Community woman autonomy status**				
Low			1.96*** [1.58,2.41]	1.66***[1.26, 2.19]
Moderate			1.70***[1.34, 2.15]	1.61** [1.23, 2.10]
High			**1**	**1**
**Community young age at first marriage**				
Low			**1**	**1**
Moderate			1.14 [0.94, 1.38]	1.06 [0.85, 1.31]
High			1.30* [1.06, 1.60]	1.24 [0.96, 1.59]
**Random effects**				
Variance (CI)	0.27 [0.19–0.38]	0.21 [0.12–0.36]	0.19 [0.12–0.29]	0.14 [0.07–0.28]
ICC (%)	7.6	5.9	5.5	4.1
PCV (%)	Ref	22.2	29.6	48.1
Wald chi-square	Ref	484.28***	66.31***	541.28***
**Model fitness**				
Log-likelihood	-3360.8	-2187.3	-3327.8	-2167.4
AIC	6725.6	4426.6	6681.6	4408.8
BIC	6739.4	4599.8	6771.3	4655.3
**N**	**7,358**	**7,358**	**7,358**	**7,358**

*** *p* < 0.001; ** = *p* < 0.01; * = *p* < 0.05; 1 = Reference Category; Model 0 contains no explanatory variables; Model I includes individual-level factors only; Model II includes both individual-level and community-level factors; Model III includes community-level factors only aOR adjusted odds ratio, CI confidence internal, ICC intraclass correlation coefficient, PCV Proportional change in variance, AIC Akaike information criterion, BIC Bayesian Information Criterion

## Discussion

This study sought to analyse the influence of individual and community-level factors that explain the experience of spousal physical among ever-married violence in Zambia. The study applied a multilevel logistic regression models on the recent Zambia Demographic and Health Survey conducted in 2018. Disparities in experience of spousal violence have been observed among different sociodemographic strata and a further understanding of these factors has a significant implication on strengthening strategies and programmes aimed at further reducing the prevalence of spousal violence in the country.

Our study reveals that the prevalence of ever-married women who experienced spousal physical violence in the twelve months’ period prior to the survey is Zambia. The prevalence of intimate partner violence found in this analysis is similar to what was previously reported in a study conducted by Marifa et al., [23], which identified a prevalence of spousal physical violence to be high in among ever-married in Uganda, Mali and Angola [23]. Angaw et al., also reported a high proportion of ever-married women in Ethiopia experienced intimate partner violence [27]. This finding of our study has significant implication for strengthening GBV policies and interventions to further reduce the prevalence of intimate partner violence against women in Zambia. If this situation is unattended to women will continue to experiencing severe physical injuries, mental disorders. unplanned pregnancies and exposure to HIV or other sexually transmitted illnesses [47]. Policy measures to eradicate GBV should thus focus on prevention strategies that promote gender equality through empowerment of women and girls through education. Furthermore, there is need to promote community initiatives that engage men and boys to participate in designing and implementation of GBV interventions.

This study has established that individual factors (age of a woman, ownership of mobile phone, woman decision making autonomy, number of wives; consumption of alcohol; partner display of jealous behaviour and accusation of infidelity) and community factors (woman decision making autonomy and age at first marriage) were significantly associated with an experience of physical spousal violence among ever-married women in Zambia.

Results showed that married women aged 15–19 years and 24–24 years had generally higher odds of experiencing physical spousal violence in the past 12 months compared with older women (aged 45–49 years). Literature show that prior studies conducted in SSA have not reported uniform findings in terms of the relationship between age of a woman and experience of intimate partner violence. However, our finding is consistent with studies that have indicated that as age of a woman increases, the experience of physical violence decreases [27, 29, 48, 49]. This finding could be explained by the fact that older women are more likely have decision making power and are able to seek support on personal strategies that prevent exposure to domestic violence compared to their young counterparts [49–51]. This finding has an implication for designing of community strategies prevent early marriages and empower young women with adequate information on how to protect themselves from spousal violence. Strategies initiated by the World bank such as the Keeping Girls in school project which is aimed at enhancing community access to education should be rolled out to the whole country in a bid to prevent young girls from falling into the trap of early marriage [52]. Furthermore, educating boys and men about gender equality to enable them treat women as full human beings in their own right is key to reducing the prevalence of early marriages in communities. Additionally, engaging of community influential leaders is key in achieving the goal to reduce child marriages in many cultures that practice early marriage, as they have the power to affect the way social norms are practiced in small communities [53].

The study has shown that women in Zambia who own mobile phones were significantly less likely to experience spousal physical violence compared to those who did not own mobile phones. This finding indicates that having a mobile phone may facilitate the empowerment of women by exposing them to opportunities for economic and networking growth, political participation, and skill development [54], thereby reducing financial dependence of the women on their spouses [54]. This finding has significant implication on promotion of ownership of ICT devices as information tools on human rights, wellbeing and empowerment.

Our study revealed that women who had decision-making autonomy regarding household purchases, own health, and visiting to family and relative had reduced likelihood of experiencing spousal physical violence. This finding is consistent with findings reported by prior studies conducted in other settings in SSA [9, 27, 29, 55, 56]. This finding may have a number of potential explanations, one of which is that women who have autonomy in decision-making are better able to advocate for their rights and challenge some of the choices made by their spouses. This suggest that empowering women has the potential to reduce women’s exposure to domestic violence in Zambia and in SSA in general.

This study established that experience of spousal physical violence was observed to be higher among women whose partners were drinking alcohol compared to women whose partners were not consuming alcohol. The studies that were carried out in Tanzania, Nigeria, Uganda, Ethiopia, Ghana, and Malawi all point to the same conclusion, which is supported by these findings [31, 57–61]. The effect of alcohol on men’s cognitive capacities, reduced self-control and heightened patriarchal ideas, which in turn arouse toxic masculinities, could be one of the possible explanation of the finding of this study. Alcohol consumption can make men more aggressive and less able to negotiate a peaceful conclusion to a conflict within the partnership. Additionally, excessive drinking can lead to financial problems and make other family concerns worse. This may lead to marital conflict and tension, which raises the possibility of violence [9, 31, 62]. The present study has also revealed that women whose partners were jealous as well as those who were being accused of being unfaithful were equally more likely to be abused by their partners. Similar results were reported in a study conducted in Zimbabwe [55].

Differences in experience of spousal physical violence were observed according to distinct individual and community-level factors. Therefore, enhancing women empowerment through access to education for female adolescents, creating employment opportunities for women and strengthening of sexual reproductive programme interventions to discourage early marriages will be key to addressing the problem of gender based violence against women in Zambia. As evidenced by the results, women who have decision making autonomy were less susceptible to spousal physical violence, suggesting that empowering women could go a long way in addressing intimate partner violence against girls and women.

There could be unobserved or unmeasured community-level factors that influenced spousal physical violence in Zambia as evidenced by the intra-class correlation coefficient in the full regression model. This suggests that there could be factors operating at the community-level, not included in this current analysis, which may be associated with experience of spousal physical violence in Zambia. These may include, but are not limited to, cultural differences between communities (that may ultimately influence intimate partner violence). Therefore, further interventions to curb intimate partner violence will require community profiling to understand the norms and cultural values that perpetuate gender based violence. Furthermore, community engagement among relevant stakeholders such as civic leaders, traditional leaders, community leaders and religious institutions can play a leading role in engaging men to participate in coming up with and implementation of community led actions aimed at preventing women from risks associated with intimate partner violence.

This study has provided useful findings that have the potential to inform strengthening of existing policies, strategies and programmes aimed at reducing GBV against women in Zambia. However, designing of context specific interventions to address the problem will require a detailed decomposition analysis of both individual and community-level factors to delineate the contribution effects of various factors to trends in GBV rates in the country.

## Study strengths and limitations

The study had a number of limitations. First, because of the cross-sectional nature of the DHS data, causality cannot be inferred from this study. Second, the outcome of interest intimate partner violence was measured for the 12 months’ period prior to the survey. But the independent factors are with reference to the time when the survey was conducted, meaning that there is a possibility of a variance between some factors at the time of the event happened and those at the time of the survey. There is also a possibility of recall bias, since the DHS participants were asked to report events that happened in the past. Since the study comprised a nationally representative sample of Zambian women aged 15–49 years, the current findings can apply to the entire population of ever-married women in Zambia. The hierarchical nature of the DHS dataset allowed for exploration of community effects, which may have an influence on gender-based violence programming in the Zambian context. The study also assessed a wide range of factors to strengthen the associations observed between experience of spousal physical violence and women’s individual and contextual factors.

## Conclusion

In Zambia, slightly over one-fifth of women of ever-married of reproductive age experienced spousal physical violence. A women’s age, ownership of mobile phone, women’s decision-making autonomy, partner’s alcohol consumption status, partner’s display of jealous behaviour, partner accusation of infidelity were the major determinants of spousal physical violence. Promoting access to mobile technologies would be key in enhancing access to and utilisation of information forums that may help women to prevent intimate partner violence. Further, we recommend designing and implementing of community level women’s empowerment strategies through education and employment opportunities to increase the proportion of women who have decision-making autonomy at household and societal level. Effective couple counselling will be key to building trust between wife and husband. Integrating community level interventions aimed at breaking the societal tolerance towards IPV will in the long run eliminate the negative norms and culture that predispose women to IPV.

## Data Availability

Data used in our study is publicly available at DHS program website (https://dhsprogram.com/).

## List of Abbreviations

CI: Confidence Interval
EA: Enumeration Area
GBV: Gender-Based Violence
IPV: Intimate Partner Violence
SDG: Sustainable Development Goal
SRH: Sexual Reproductive Health
SSA: Sub-Saharan Africa
UNFPA: United Nations Population Fund
USAID: United States Aid for International Development
WHO: World Health Organisation
ZDHS: Zambia Demographic and Health Survey

## References

[1] García-Moreno C, Pallitto C, Devries K, Stöckl H, Watts C, Abrahams N. Global and regional estimates of violence against women: prevalence and health effects of intimate partner violence and non-partner sexual violence. World Health Organization; 2013.

[2] Wado YD, Mutua MK, Mohiddin A, Ijadunola MY, Faye C, Coll CVN et al. Intimate partner violence against adolescents and young women in sub-saharan Africa: who is most vulnerable? Reproductive Health 2021 Jun 17;18(1):119. 10.1186/s12978-021-01077-z PMC8210343 34134704

[3] Enaifoghe A, Dlelana M, Durokifa AA, Dlamini NP. The prevalence of gender-based violence against women in South Africa: a call for action. African Journal of gender, Society and Development (formerly Journal of gender, information and development in Africa). 2021 Mar;10(1):117–46.

[4] Zinyemba KG, Hlongwana K. Men’s conceptualization of gender-based violence directed to women in Alexandra Township, Johannesburg, South Africa. BMC Public Health 2022 Nov 30;22(1):2235. 10.1186/s12889-022-14616-5 PMC9713989 36451124

[5] Simona S, Muchindu M, Ntalasha H (2018). Intimate Partner Violence (IPV) in Zambia: Socio-demographic determinants and association with use of maternal Health Care. Int’l J Soc Sci Stud.

[6] Yount KM, Cheong YF, Khan Z, Bergenfeld I, Kaslow N, Clark CJ. Global measurement of intimate partner violence to monitor sustainable development goal 5. BMC Public Health. 2022 Mar;8(1):465. 10.1186/s12889-022-12822-9 PMC8903149 35260134

[7] Le Port A, Seye M, Heckert J, Peterman A, Nganya Tchamwa A, Dione M et al. A community edutainment intervention for gender-based violence, sexual and reproductive health, and maternal and child health in rural Senegal: a process evaluation. BMC Public Health 2022 Jun 10;22(1):1165. 10.1186/s12889-022-13570-6 PMC9185706 35689180

[8] World Health Organisation and Pan American Health Organisation (2012). Understanding and addressing violence against women.

[9] Kebede SA, Weldesenbet AB, Tusa BS. Magnitude and determinants of intimate partner violence against women in East Africa: multilevel analysis of recent demographic and health survey. BMC Women’s Health. 2022 Dec;22(1):74. 10.1186/s12905-022-01656-7 PMC8928594 35300675

[10] World Health Organization (2019). Violence against women intimate partner and sexual violence against women [Internet].

[11] World Health Organization (2017). Violence against women: key facts. World Health Organization Last modified November.

[12] Kishor S, Johnson K (2005). Profiling domestic violence: a multi-country study. Stud Fam Plann.

[13] Hindin MJ, Kishor S, Ansara DL. Intimate partner violence among couples in 10 DHS countries: predictors and health outcomes. Macro International Incorporated; 2008.

[14] Vives-Cases C, Ruiz-Cantero MT, Escribà-Agüir V, Miralles JJ. The effect of intimate partner violence and other forms of violence against women on health. Journal of Public Health. 2011 Mar 1;33(1):15–21. 10.1093/pubmed/fdq101 21196478

[15] Maxwell L, Devries K, Zionts D, Alhusen JL, Campbell J. Estimating the Effect of Intimate Partner Violence on Women’s Use of Contraception: A Systematic Review and Meta-Analysis. PLOS ONE. 2015 Feb 18;10(2):e0118234. 10.1371/journal.pone.0118234 PMC4334227 25693056

[16] Maxwell L, Nandi A, Benedetti A, Devries K, Wagman J, García-Moreno C. Intimate partner violence and pregnancy spacing: results from a meta-analysis of individual participant time-to-event data from 29 low-and-middle-income countries. BMJ Glob Health. 2018 Jan;3(1):e000304. 10.1136/bmjgh-2017-000304 PMC5859805 29564152

[17] SUTHERLAND CA, SULLIVAN CM. BYBEE DI. Effects of Intimate Partner Violence Versus Poverty on Women’s Health. Violence Against Women. 2001 Oct 1;7(10):1122–43.

[18] Geffner R. The Effects of Intimate Partner Violence on Children [Internet]. Routledge; 2014 [cited 2022 Dec 28]. Available from: https://www.taylorfrancis.com/books/mono/10.4324/9781315044026/effects-intimate-partner-violence-children-robert-geffner-robyn-spurling-igelman-jennifer-zellner.

[19] Staggs SL, Riger S. Effects of intimate Partner violence on low-income women’s Health and Employment. Am J Community Psychol. 2005 Sep;36(1):133–45. 10.1007/s10464-005-6238-1 16134050

[20] Izugbara CO, Obiyan MO, Degfie TT, Bhatti A. Correlates of intimate partner violence among urban women in sub-saharan Africa. PLoS ONE. 2020 Mar;25(3):e0230508. 10.1371/journal.pone.0230508 PMC7094863 32210457

[21] Gubi D, Nansubuga E, Wandera SO. Correlates of intimate partner violence among married women in Uganda: a cross-sectional survey. BMC Public Health 2020 Jun 26;20(1):1008. 10.1186/s12889-020-09123-4 PMC7318470 32586297

[22] McCloskey LA, Boonzaier F, Steinbrenner SY, Hunter T. Determinants of intimate Partner violence in Sub-Saharan Africa: a review of Prevention and intervention programs. Partn Abuse 2016 Jan 1;7(3):277–315.

[23] Muchemwa M, Phiri M, Aladejebi O. Information communication technologies and intimate partner violence among women in sub-Saharan Africa countries. 2022 Sep 15 [cited 2022 Sep 30]; Available from: https://dhsprogram.com/publications/publication-WP186-Working-Papers.cfm.

[24] Bolarinwa OA, Ahinkorah BO, Frimpong JB, Seidu AA, Tessema ZT. Spatial distribution and predictors of intimate partner violence among women in Nigeria. BMC Women’s Health. 2022 Dec;22(1):245. 10.1186/s12905-022-01823-w PMC9208710 35725404

[25] Ahinkorah BO. Intimate partner violence against adolescent girls and young women and its association with miscarriages, stillbirths and induced abortions in sub-saharan Africa: evidence from demographic and health surveys. SSM - Population Health. 2021 Mar;1:13:100730. 10.1016/j.ssmph.2021.100730 PMC7815812 33511264

[26] Tantu T, Wolka S, Gunta M, Teshome M, Mohammed H, Duko B. Prevalence and determinants of gender-based violence among high school female students in Wolaita Sodo, Ethiopia: an institutionally based cross-sectional study. BMC Public Health. 2020 Apr 21;20(1):540. 10.1186/s12889-020-08593-w PMC7345512 32316941

[27] Angaw DA, Melesse AW, Geremew BM, Tesema GA. Spatial distribution and determinants of intimate partner violence among reproductive-age women in Ethiopia: spatial and multilevel analysis. BMC Womens Health 2021 Feb 25;21(1):81. 10.1186/s12905-021-01218-3 PMC7905923 33632206

[28] Cofie N. A multilevel analysis of contextual risk factors for intimate partner violence in Ghana. Int Rev Victimology. 2020 Jan;26(1):50–78.

[29] Tiruye TY, Harris ML, Chojenta C, Holliday E, Loxton D (2020). Determinants of intimate partner violence against women in Ethiopia: a multi-level analysis. PLoS ONE.

[30] Seid E, Melese T, Alemu K. Spatial distribution and predictors of domestic violence against women: evidence from analysis of ethiopian demographic health survey 2016. BMC Women’s Health 2021 Sep 15;21(1):334. 10.1186/s12905-021-01465-4 PMC8442426 34525981

[31] Benebo FO, Schumann B, Vaezghasemi M. Intimate partner violence against women in Nigeria: a multilevel study investigating the effect of women’s status and community norms. BMC Women’s Health 2018 Aug 9;18(1):136. 10.1186/s12905-018-0628-7 PMC6085661 30092785

[32] Corsi DJ, Neuman M, Finlay JE, Subramanian S. Demographic and health surveys: a profile. International Journal of Epidemiology. 2012 Dec 1;41(6):1602–13. 10.1093/ije/dys184 23148108

[33] Black E, Worth H, Clarke S, Obol JH, Akera P, Awor A, et al. Prevalence and correlates of intimate partner violence against women in conflict affected northern Uganda: a cross-sectional study. Confl Health. 2019 Jul;30(1):35. 10.1186/s13031-019-0219-8 PMC6668065 31384294

[34] Ward CL, Harlow S. R.E.S.P.e.c.T and intimate partner violence: a cross-sectional study using DHS data in Kenya. BMJ Open. 2021 Sep 1;11(9):e046069. 10.1136/bmjopen-2020-046069 PMC8424846 34493507

[35] Croft TN, Aileen MJM, Courtney KA (2018). Guide to DHS Statistics.

[36] Wado YD, Gurmu E, Tilahun T, Bangha M. Contextual influences on the choice of long-acting reversible and permanent contraception in Ethiopia: a multilevel analysis. Haider MR. editor PLoS ONE. 2019 Jan;16(1):e0209602. 10.1371/journal.pone.0209602 PMC6334991 30650085

[37] Ahinkorah BO, Seidu AA, Appiah F, Budu E, Adu C, Aderoju YBG, et al. Individual and community-level factors associated with modern contraceptive use among adolescent girls and young women in Mali: a mixed effects multilevel analysis of the 2018 Mali demographic and health survey. Contracept Reprod Med. 2020 Dec;5(1):27. 10.1186/s40834-020-00132-7 PMC7547459 33062298

[38] Lasong J, Zhang Y, Gebremedhin SA, Opoku S, Abaidoo CS, Mkandawire T et al. Determinants of modern contraceptive use among married women of reproductive age: a cross-sectional study in rural Zambia. BMJ Open. 2020 Mar 1;10(3):e030980. 10.1136/bmjopen-2019-030980 PMC7170561 32234737

[39] Fenta SM, Gebremichael SG. Predictors of modern contraceptive usage among sexually active rural women in Ethiopia: a multi-level analysis. Arch Public Health. 2021 Dec;79(1):93. 10.1186/s13690-021-00621-4 PMC8176723 34088347

[40] Ahinkorah BO. Predictors of modern contraceptive use among adolescent girls and young women in sub-saharan Africa: a mixed effects multilevel analysis of data from 29 demographic and health surveys. Contracept Reproductive Med. 2020 Nov;19(1):32. 10.1186/s40834-020-00138-1 PMC7678092 33292675

[41] Aychiluhm SB, Tesema AK, Tadesse AW. Early marriage and its determinants among Married Reproductive Age Group Women in Amhara Regional State, Ethiopia: a Multilevel Analysis. Biomed Res Int. 2021 Mar 9;2021:e1969721. 10.1155/2021/1969721 PMC7963895 33763468

[42] Misunas C, Erulkar A, Apicella L, Ngô T, Psaki S. What influences girls’ age at marriage in Burkina Faso and Tanzania? Exploring the contribution of Individual, Household, and Community Level factors. J Adolesc Health. 2021 Dec;69(6):46–56. 10.1016/j.jadohealth.2021.09.015 34809900

[43] Phiri M, Musonda E, Shasha L, Kanyamuna V, Lemba M. Individual and community-level factors associated with early marriage in Zambia: a mixed effect analysis. BMC Women’s Health 2023 Jan 17;23(1):21. 10.1186/s12905-023-02168-8 PMC9843915 36650478

[44] Diez-Roux AV (2000). Multilevel analysis in Public Health Research. Annu Rev Public Health.

[45] Merlo J, Wagner P, Ghith N, Leckie G. An Original Stepwise Multilevel Logistic Regression Analysis of Discriminatory Accuracy: The Case of Neighbourhoods and Health. Moerbeek M, editor. PLoS ONE. 2016 Apr 27;11(4):e0153778. 10.1371/journal.pone.0153778 PMC4847925 27120054

[46] Goldstein H. Multilevel Statistical Models. John Wiley & Sons; 2011. p. 376.

[47] Wirtz AL, Perrin NA, Desgroppes A, Phipps V, Abdi AA, Ross B et al. Lifetime prevalence, correlates and health consequences of gender-based violence victimisation and perpetration among men and women in Somalia. BMJ Global Health. 2018 Jul 1;3(4):e000773. 10.1136/bmjgh-2018-000773 PMC6074632 30105094

[48] Pathak N, Dhairyawan R, Tariq S (2019). The experience of intimate partner violence among older women: a narrative review. Maturitas.

[49] Mezey NJ, Post LA, Maxwell CD. Redefining intimate partner violence: women’s experiences with physical violence and non-physical abuse by age. International Journal of Sociology and Social Policy. 2002 Jan 1;22(7/8):122–54.

[50] Lundy M, Grossman SF. Domestic violence service users: a comparison of older and younger women victims. J Fam Viol. 2009 Jul;24(1):297–309.

[51] Beaulaurier RL, Seff LR, Newman FL, Dunlop B. Internal barriers to help seeking for Middle-Aged and older women who experience intimate Partner violence. J Elder Abuse Negl 2005 Aug 19;17(3):53–74. 10.1300/j084v17n03_04 16931469

[52] World Bank (2015). Adolescent girls in Zambia, policy brief.

[53] Mehra D, Sarkar A, Sreenath P, Behera J, Mehra S. Effectiveness of a community based intervention to delay early marriage, early pregnancy and improve school retention among adolescents in India. BMC Public Health 2018 Jun 14;18(1):732. 10.1186/s12889-018-5586-3 PMC6000967 29898696

[54] Cardoso LF, Sorenson SB (2017). Violence against women and household ownership of radios, computers, and phones in 20 countries. Am J Public Health.

[55] Bengesai AV, Khan HTA. Female autonomy and intimate partner violence: findings from the Zimbabwe demographic and health survey, 2015. Cult Health Sex. 2021 Jul;23(7):927–44. 10.1080/13691058.2020.1743880 32285753

[56] Zegenhagen S, Ranganathan M, Buller AM. Household decision-making and its association with intimate partner violence: examining differences in men’s and women’s perceptions in Uganda. SSM - Population Health. 2019 Aug;1:8:100442. 10.1016/j.ssmph.2019.100442 PMC6612922 31321280

[57] Vyas S, Jansen HAFM. Unequal power relations and partner violence against women in Tanzania: a cross-sectional analysis. BMC Women’s Health 2018 Nov 15;18(1):185. 10.1186/s12905-018-0675-0 PMC6238293 30442127

[58] Amegbor PM, Pascoe L. Variations in emotional, sexual, and physical intimate Partner Violence among Women in Uganda: a Multilevel Analysis. J Interpers Violence. 2021 Aug;36(15–16):NP7868–98. 10.1177/0886260519839429 30924708

[59] Liyew AM, Alem AZ, Ayalew HG. Magnitude and factors associated with intimate partner violence against pregnant women in Ethiopia: a multilevel analysis of 2016 ethiopian demographic and health survey. BMC Public Health. 2022 Feb;11(1):284. 10.1186/s12889-022-12720-0 PMC8840032 35148725

[60] Ogum Alangea D, Addo-Lartey AA, Sikweyiya Y, Chirwa ED, Coker-Appiah D, Jewkes R et al. Prevalence and risk factors of intimate partner violence among women in four districts of the central region of Ghana: Baseline findings from a cluster randomised controlled trial. PLoS One. 2018 Jul 19;13(7):e0200874. 10.1371/journal.pone.0200874 PMC6053193 30024948

[61] Chilanga E, Collin-Vezina D, Khan MN, Riley L. Prevalence and determinants of intimate partner violence against mothers of children under-five years in Central Malawi. BMC Public Health. 2020 Dec;2(1):1848. 10.1186/s12889-020-09910-z PMC7709392 33267864

[62] Shakya HB, Cislaghi B, Fleming P, Levtov RG, Boyce SC, Raj A et al. Associations of attitudes and social norms with experiences of intimate partner violence among married adolescents and their husbands in rural Niger: a dyadic cross-sectional study. BMC Women’s Health. 2022 May 18;22(1):180. 10.1186/s12905-022-01724-y PMC9118706 35585589

